# Coronary Fistula Between the Left Anterior Descending Coronary Artery and the Pulmonary Artery: A Case Report

**DOI:** 10.1002/ccr3.71418

**Published:** 2025-11-09

**Authors:** Mohammad Qaiser Aziz Khan, Iman Muhammad Tahir, Vijay Kumar, Marium Nastaeen Sarhandi, Ariful Haque

**Affiliations:** ^1^ Department of Cardio‐Thoracic Surgery Liaquat National Hospital and Medical College Karachi Pakistan; ^2^ Department of Public Health Atish Dipankar University of Science and Technology Dhaka Bangladesh; ^3^ Voice of Doctors Research School Dhaka Bangladesh; ^4^ Department of Orthopedic Surgery Yan'an Hospital Affiliated to Kunming Medical University Kunming Yunnan China

**Keywords:** angina, case report, coronary vessel fistula, pulmonary artery, surgical repair, syncope

## Abstract

Timely diagnosis and management of coronary artery fistulas are vital to prevent complications. Advanced imaging and multidisciplinary care aid accurate treatment. Clinicians should consider coronary artery fistula in unexplained cardiac symptoms. When resources are limited, innovation like intraoperative fluoroscopy can ensure success. Always seek clarity when in doubt to guide clinical decisions.

## 1 Introduction

Coronary artery fistula (CAF) is a rare disease of the coronary circulation system. These malformations are usually congenital, but over the years, acquired cases are observed after cardiac surgery, myocardial biopsy, direct chest trauma, or secondary to conditions like infective endocarditis and neoplasms [1]. It affects the assembly and function of coronary arteries, which occur in about 0.9% of the population [2]. They are usually diagnosed incidentally on coronary angiography and noninvasive tests like ECG, treadmill test, and echocardiography. The right coronary artery (RCA) is the most common source of origin, followed by the left coronary artery. The most common insertion sites in descending order, are the right ventricle (RV), right atrium (RA), and the pulmonary arteries. However, recent findings suggest that the most frequent fistula involves the left main artery or the left anterior descending (LAD) artery and the pulmonary arteries [3]. The management of CAF is still questionable in terms of appropriate intervention and invasive versus noninvasive approach. Due to the complexity and rarity of this anomaly, each patient requires a personalized management approach. In this case, a combination of surgical and medical treatment was successfully implemented at our private tertiary care hospital, ensuring optimal patient outcomes.

## 2 Case History

A 39‐year‐old male, a smoker, with treated hepatitis C, presented to a tertiary care hospital in Karachi, Pakistan with complaints of retrosternal chest pain radiating to the left arm. The pain was aggravated by exertion and was associated with progressive shortness of breath. He experienced multiple episodes of loss of consciousness. His shortness of breath occurred with minimal activity and worsened to the point where he could no longer perform daily activities. He reported a history of similar intermittent episodes for several years.

Upon admission, physical examination revealed a middle‐aged male with a blood pressure of 150/90 mmHg and a heart rate of 89 beats per minute. His oxygen saturation was 98% on room air. Auscultation of the heart revealed normal heart sounds. Troponin I was done to rule out myocardial infarction and was normal < 0.10.

## 3 Methods

**FIGURE 1:**
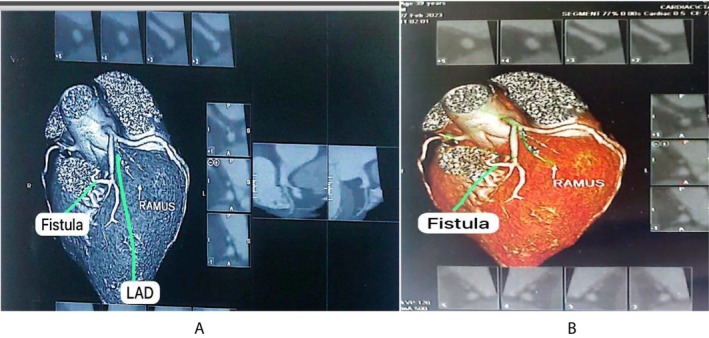
(A and B) Preoperative CT‐angiogram showing an abnormal tortuous branch from mid LAD forming an abnormal fistulous connection with the main pulmonary artery.

**FIGURE 2:**
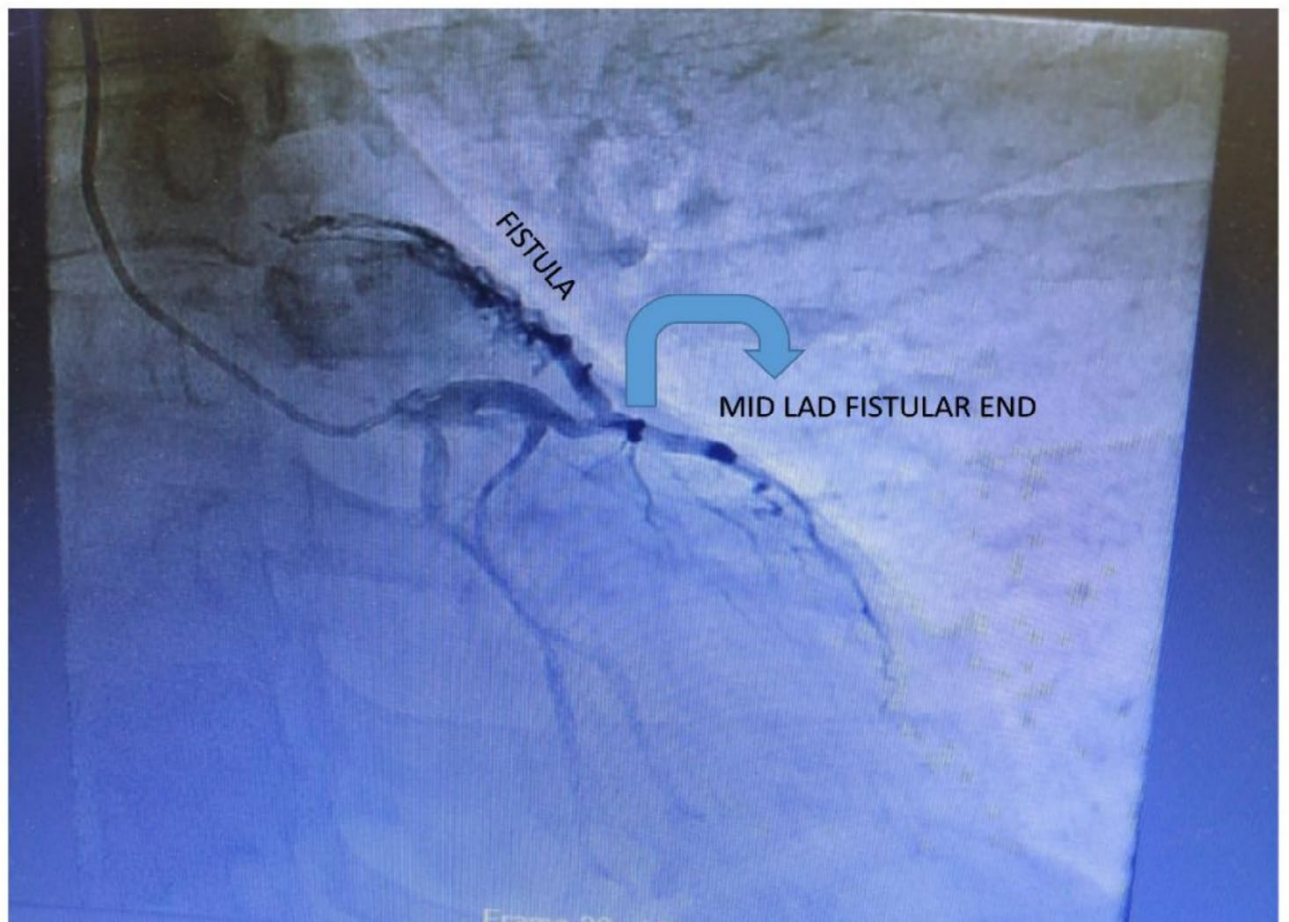
Preoperative angiography showing fistula between LAD and main pulmonary artery.

His electrocardiogram demonstrated sinus rhythm. Trans‐thoracic echocardiography showed normal left ventricle (LV) size with normal LV systolic dysfunction, thickened mitral valve and atrial valve with normal mobility, normal RV systolic function, and 55% ejection fraction (EF). Tricuspid Annular Plane Systolic Excursion (TAPSE) was 18 mm. Later, the patient became a candidate for coronary angiography and underwent coronary CT angiography (CTA), which revealed an abnormal tortuous branch arising from the proximal segment of the LAD adjacent to the level of origin of the D1 branch. It showed abnormal fistulous communication with the main pulmonary trunk (Figures 1 and 2). LAD and RCA showed minimal plaque without significant stenosis. OM (obtuse marginal) and RCA showed a 20%–30% lesion.

In this case report, the patient underwent a median sternotomy to access the pericardial cavity, aiming to address a suspected fistula involving the proximal LAD. Sharp dissection of the epicardium revealed four branches emanating from the LAD: diagonal, septal, suspected fistula, and distal LAD branches. Based on anatomical positioning, one vessel suspected to be the fistulous connection was clipped during the procedure. Following the closure of the chest, the patient was moved to the Surgical Intensive Care Unit (SICU). However, due to persistent hemodynamic instability, a decision was made to conduct a review angiography in the catheterization lab.

During angiography, a residual fistula opening at the LAD end was confirmed, (Video 1), prompting the patient's return to the operating room. Under the guidance of angiography images, the LAD end of the fistula was identified and closed using 5/0 prolene sutures. To ensure complete closure, the cardiology team performed fluoroscopy in the operating room, confirming successful closure at both ends of the fistula. Intraoperative blood samples drawn directly from the pulmonary artery for arterial blood gases (ABGs) showed significant improvement post‐closure, with the post‐closure partial pressure of oxygen (PO2) rising to 259 mmHg from a pre‐closure value of 76.9 mmHg.

**VIDEO 1:** Intra‐operative angiography showing residual fistula opening at LAD. Video content can be viewed at https://onlinelibrary.wiley.com/doi/10.1002/ccr3.71418.

**FIGURE 3:**
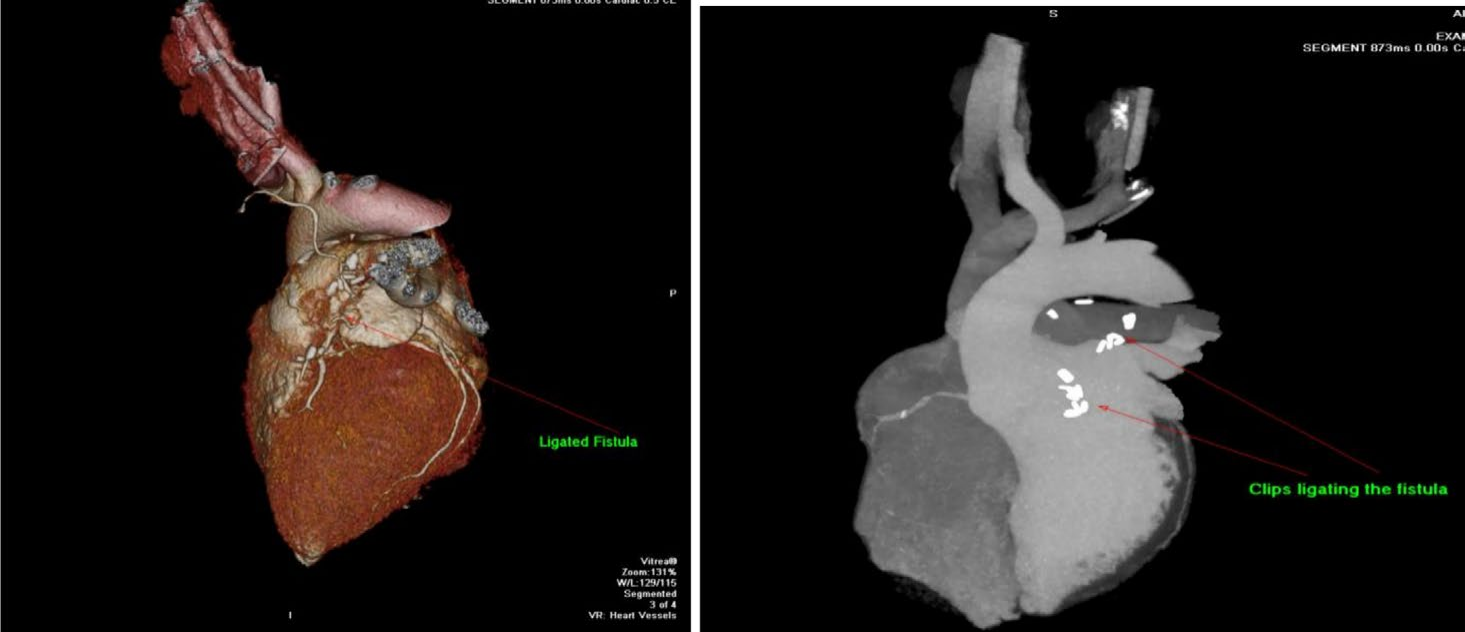
Postoperative CT coronary angiogram showing ligated fistula.

After these interventions, the patient had an uncomplicated recovery in the ICU and was discharged on the fourth day postoperatively. On his recent follow‐up visit 9 months post‐surgery, he had no active complaints. His echocardiogram was repeated, and it showed an EF of 45% and normal LV size with mildly reduced LV function. A CTA was done that revealed mild < 50% disease in LAD and Diagonal (Figure 3).

This case underscores the complexity and challenges in managing cardiac fistulas, emphasizing the critical role of multidisciplinary collaboration between surgical and cardiology teams for accurate diagnosis, intervention, and postoperative care in such cases.

## 4 Discussion

CAF is a rare vascular anomaly characterized by an abnormal connection between a coronary artery and a cardiac chamber, coronary sinus, superior vena cava, pulmonary artery, or pulmonary vein, leading to altered hemodynamics and potential clinical complications [3]. It is one of the rare cardiac congenital anomalies representing 0.9% of total cases of congenital cardiac anomalies [1]. Most (50%) of the fistulas were found to originate from the RCA. In 42% of cases, it originates from the LCA while in 5% of cases, it originates from both coronary arteries. The RV (41%) is the most frequent site of drainage followed by the RA (26%), and the pulmonary artery (17%) [4]. The majority of cases are primary congenital anomalies, but they can be acquired. The causes of acquired cases are coronary artery bypass graft, trauma, and Takayasu arteritis [5].

CAFs are further subdivided into coronary‐cameral fistulas and coronary IV fistulas. Improper communication between a coronary artery and one of the heart chambers is commonly referred to as a coronary cameral fistula. A coronary intravenous fistula is an abnormal arterial connection between the coronary artery and the systemic or pulmonary circulation [6].

Clinically CAF is asymptomatic in adult patients. In comparison with adults, a lesser percentage of pediatric patients are asymptomatic. It was rarely diagnosed in infants [3]. Symptomatically it presents as dyspnea, angina with exertion, and occasionally arrhythmias. The presence of a continuous pericardial murmur on physical examination is highly suggestive of high‐flow CAFs. If CAF remains untreated, it can lead to complications such as coronary steal syndrome, rupture, endocarditis, and atrial fibrillation [7].

In the diagnosis of CAF, coronary angiography plays a vital role while coronary computed tomography angiography (CCTA) delineates the path of the fistula more accurately and noninvasively [5]. In a previous case report, such as those by Dadkhah‐Tirani H et al. (2013), it has highlighted the importance of CCTA in detecting fistulous connections [4]. In our case, CCTA was instrumental in confirming the diagnosis and guiding surgical planning. In suspected cases, transesophageal echocardiography (TEE) is helpful. It enhances the heart's morphology and the anatomy of the fistula and also significantly locates the CAF drainage site preoperatively. Additionally, other diagnostic methods such as computed tomography (CT) and magnetic resonance imaging (MRI) are useful to assess the precise anatomy of a CAF [8]. However, one key limitation associated with CT scans is radiation exposure, which must be taken into account when making imaging recommendations [6]. MRI, an alternative to CT, offers more detailed information than standard contrast angiography. It provides enhanced anatomical imaging that defines the origin and outflow site of CAFs [9].

The guidelines of the American College of Cardiology and the American Heart Association for the management of adults with congenital heart disease state that large CAF should be closed regardless of symptoms. In contrast, the small or medium‐sized fistula should only be closed if it is symptomatic [9]. The invasive procedure for the CAF can be either catheter‐guided coil embolization or surgical occlusion of the fistula which can be either catheter‐guided coil embolization or surgical occlusion of the fistula. In our case, a surgical approach was undertaken as the anatomy of the fistula was complex, which was supported by studies like George V et al. (2022) [6]. To ensure complete closure, we adopted an innovative strategy by utilizing the C‐Arm available in our operating room to perform on‐table fluoroscopy. This allowed real‐time confirmation of fistula closure through contrast injection, demonstrating that fluoroscopy can be a valuable intraoperative tool even in resource‐constrained settings. Our experience highlights the importance of adaptability and innovation in clinical practice, especially when standard resources may be limited. Clinicians should maintain a high index of suspicion for coronary artery fistulas in patients with unexplained angina, syncope, or other atypical cardiac symptoms, and when diagnostic uncertainty exists, it is crucial to seek clarity through additional imaging and interdisciplinary collaboration.

Incomplete closure of CAF leading to hemodynamic instability can arise from several factors. Congenital fistulas, due to abnormal embryogenesis, often have complex, branching structures that complicate complete closure. Trauma from stab wounds or gunshots may cause irregular fistulas with damaged tissues that are difficult to seal. Invasive procedures, such as coronary angiography or pacemaker implantation, can result in iatrogenic fistulas, where weakened tissues may lead to incomplete repair. Additionally, cardiac surgeries like septal myomectomy can inadvertently create fistulas or complicate closures due to scarring or tissue damage. In all cases, residual fistulas can allow abnormal blood flow to persist, leading to continued hemodynamic instability.

## 5 Conclusion

Our case serves as a good example of a rare congenital anomaly and its appropriate management according to the course of the disease. Although rare, this diagnosis should be considered for all patients presenting with symptoms such as angina, syncope, and orthopnea, as demonstrated by this patient.

## Author Contributions

**Mohammad Qaiser Aziz Khan:** conceptualization, supervision, writing – review and editing. **Iman Muhammad Tahir:** writing – original draft, writing – review and editing. **Vijay Kumar:** writing – original draft, writing – review and editing. **Marium Nastaeen Sarhandi:** supervision, writing – original draft, writing – review and editing. **Ariful Haque:** visualization, writing – review and editing.

## Ethics Statement

Our institution does not require ethical approval to report individual cases or case series.

## Consent

Written informed consent was acquired from the patient whose clinical images and case details are written in the study to publish this report in accordance with the journal's patient consent policy.

## Conflicts of Interest

The authors declare no conflicts of interest.

## Data Availability

The datasets used during the study are available from the corresponding author up on reasonable request.

## References

[1] D. Buccheri , “Coronary Artery Fistulae,” Arquivos Brasileiros de Cardiologia 117, no. 1 (2021): 89–90. 34320075 10.36660/abc.20210501 PMC8294720

[2] J. J. Lim , J. I. Jung , B. Y. Lee , and H. G. Lee , “Prevalence and Types of Coronary Artery Fistulas Detected With Coronary CT Angiography,” AJR American Journal of Roentgenology 203, no. 3 (2014): W237–W243. 25148179 10.2214/AJR.13.11613

[3] N. A. Zenooz , R. Habibi , L. Mammen , J. P. Finn , and R. C. Gilkeson , “Coronary Artery Fistulas: CT Findings,” Radiographics: A Review Publication of the Radiological Society of North America, Inc 29, no. 3 (2009): 781–789. 19448115 10.1148/rg.293085120

[4] H. Dadkhah‐Tirani , A. Salari , S. Shafighnia , S. F. Hosseini , and M. Naghdipoor , “Coronary Artery to Pulmonary Artery Fistula,” American Journal of Case Reports 14 (2013): 486–488. 24298301 10.12659/AJCR.889416 PMC3843600

[5] M. A. Raufi and A. S. Baig , “Coronary Artery Fistulae,” Reviews in Cardiovascular Medicine 15, no. 2 (2014): 152–157. 25051132 10.3909/ricm0670

[6] V. George , S. Omerovic , M. Madala , and A. Kang , “Left Anterior Descending Artery to Pulmonary Artery Fistula: A Case Report,” Cureus 14, no. 7 (2022): e26713. 35821734 10.7759/cureus.26713 PMC9271271

[7] A. Al‐Douri , A. Cedars , and D. Tran , “Coronary Artery Fistula Between the Left Anterior Descending Artery and Pulmonary Artery,” Proceedings (Baylor University Medical Center) 31, no. 1 (2018): 64–66. 29686557 10.1080/08998280.2017.1401380 PMC5903525

[8] D. Buccheri , P. R. Chirco , S. Geraci , G. Caramanno , and B. Cortese , “Coronary Artery Fistulae: Anatomy, Diagnosis and Management Strategies,” Heart, Lung & Circulation 27, no. 8 (2018): 940–951. 10.1016/j.hlc.2017.07.014 29503240

[9] Y. Chen , L. Lin , Q. Deng , et al., “Coronary Artery‐Bronchial Artery Fistula Imaging Characteristics and Its Correlation With Pulmonary Disease Severity,” Heart and Vessels 37, no. 12 (2022): 2101–2106. 35729428 10.1007/s00380-022-02106-y

